# Bupivacaine-induced apoptosis independently of WDR35 expression in mouse neuroblastoma Neuro2a cells

**DOI:** 10.1186/1471-2202-13-149

**Published:** 2012-12-10

**Authors:** Misako Harato, Lei Huang, Fumio Kondo, Koji Tsunekawa, Guo-Gang Feng, Jun-Hua Fan, Naohisa Ishikawa, Yoshihiro Fujiwara, Shoshiro Okada

**Affiliations:** 1 Department of Anesthesiology, Aichi Medical University School of Medicine, Nagakute, Aichi, 480-1195, Japan; 2 Department of Pharmacology, Aichi Medical University School of Medicine, Nagakute, Aichi, 480-1195, Japan

**Keywords:** WDR35, Neuro2a cells, Bupivacaine, Reactive oxygen species, p38 MAPK

## Abstract

**Background:**

Bupivacaine-induced neurotoxicity has been shown to occur through apoptosis. Recently, bupivacaine was shown to elicit reactive oxygen species (ROS) production and induce apoptosis accompanied by activation of p38 mitogen-activated protein kinase (MAPK) in a human neuroblastoma cell line. We have reported that WDR35, a WD40-repeat protein, may mediate apoptosis through caspase-3 activation. The present study was undertaken to test whether bupivacaine induces apoptosis in mouse neuroblastoma Neuro2a cells and to determine whether ROS, p38 MAPK, and WDR35 are involved.

**Results:**

Our results showed that bupivacaine induced ROS generation and p38 MAPK activation in Neuro2a cells, resulting in apoptosis. Bupivacaine also increased WDR35 expression in a dose- and time-dependent manner. Hydrogen peroxide (H_2_O_2_) also increased WDR35 expression in Neuro2a cells. Antioxidant (EUK-8) and p38 MAPK inhibitor (SB202190) treatment attenuated the increase in caspase-3 activity, cell death and WDR35 expression induced by bupivacaine or H_2_O_2_. Although transfection of Neuro2a cells with WDR35 siRNA attenuated the bupivacaine- or H_2_O_2_-induced increase in expression of WDR35 mRNA and protein, in contrast to our previous studies, it did not inhibit the increase in caspase-3 activity in bupivacaine- or H_2_O_2_-treated cells.

**Conclusions:**

In summary, our results indicated that bupivacaine induced apoptosis in Neuro2a cells. Bupivacaine induced ROS generation and p38 MAPK activation, resulting in an increase in WDR35 expression, in these cells. However, the increase in WDR35 expression may not be essential for the bupivacaine-induced apoptosis in Neuro2a cells. These results may suggest the existence of another mechanism of bupivacaine-induced apoptosis independent from WDR35 expression in Neuro2a cells.

## Background

Bupivacaine is a sodium channel blocker administrated for local infiltration, nerve block, epidural, and intrathecal anesthesia [1]. Several clinical observations have suggested that the administration of bupivacaine in close proximity to nerves causes critical dysfunction, such as radiculopathy and paresthesia [2]. An increasing number of studies have shown that bupivacaine-induced neurotoxicity occurs through apoptosis, and it can be speculated that the administration of local anesthetics in clinical practice induces apoptotic cell death of neurons [1,3,4]. However, the mechanisms by which bupivacaine triggers neurotoxicity have not been elucidated precisely.

Reactive oxygen species (ROS) are known to stimulate a number of events and pathways that lead to apoptosis, including mitogen-activated protein kinase (MAPK) signal transduction pathways [5]. In neuronal cells, p38 MAPK, a member of the MAPK family, is preferentially activated by environmental stress and inflammatory cytokines, and it has been shown to promote neuronal cell death [6]. Recent studies have demonstrated that bupivacaine-induced apoptosis involves generation of ROS in Schwann cells [7] and activation of p38 MAPK in dorsal root ganglion (DRG) neurons [8]. Furthermore, a study using the human neuroblastoma SH-SY5Y cell line has shown that bupivacaine-induced apoptosis is associated with ROS production and activation of p38 MAPK [9].

The WD40 repeat, also known as the beta-transducin repeat or the WD, is a small structural motif of approximately 40 amino acids, typically bracketed by glycine-histidine and tryptophan-aspartate (GH-WD) [10]. Repeated WD40 motifs form a domain called the WD domain that is involved in protein-protein interactions. Proteins with WD40 repeats have important roles in a variety of cellular functions such as cell growth, proliferation, apoptosis, and intracellular signal transduction [10,11]. WD repeat-containing protein 35 (WDR35) is a novel member of this protein family [12]. Recently we reported that rat WDR35, also referred to as naofen, activates caspase-3 and promotes tumor necrosis factor (TNF)-α − induced apoptosis in HEK293 cells [13]. More recently, we reported that enhanced expression of WDR35 may mediate the activation of caspase-3 through a mitochondrial signaling pathway in lipopolysaccharide (LPS)-induced hepatocyte apoptosis [14].

Unami et al. [15] reported that both death receptor and mitochondrial signaling are involved in the process of bupivacaine-induced apoptosis in HL-60 cells. In contrast, a recent study showed that the local anesthetic lidocaine induces apoptosis via the mitochondrial pathway independently of death receptor signaling [16]. Although bupivacaine has been reported to induce cell death and critical neuronal dysfunction, the precise mechanistic cascades of these effects remain unclear. The present study was undertaken to investigate whether bupivacaine induces apoptosis in the mouse neuroblastoma Neuro2a cell line and to examine the potential involvement of WDR35, ROS, and p38 MAPK in bupivacaine-induced Neuro2a cell neurotoxicity.

## Results

### Bupivacaine induces ROS generation and p38 MAPK activation, resulting in apoptosis in Neuro2a cells

**Figure 1:**
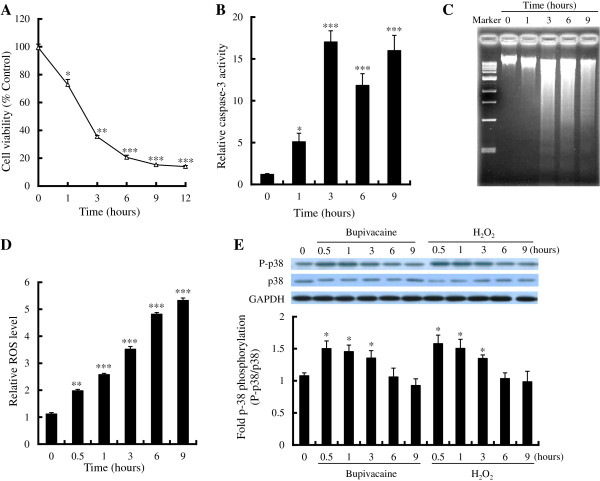
**Effect of bupivacaine on cell death, ROS generation and p38 MAPK activation.** Neuro2a cells were treated with 2 mM bupivacaine for the time indicated. (**A**) Cell viability was measured with the MTT assay. (**B**) Caspase-3 activity was measured with a fluorometric substrate. (**C**) DNA extracted from the cells was analyzed by agarose gel electrophoresis. (**D**) Intracellular levels of ROS were measured with a fluorogenic probe. (**E**) Expression of p38 and phospho-p38 (P-p38) following cell exposure to bupivacaine (2 mM) or H_2_O_2_ (0.5 mM) was measured by Western blotting. *P < 0.05, **P < 0.01 and ***P < 0.001 vs. control (not treated with bupivacaine or H_2_O_2_, n = 6).

As Werdehausen et al. reported that 2 mM bupivacaine induces apoptosis in human neuroblastoma cells [4], we first examined cell injury in 2 mM bupivacaine-treated Neuro2a cells by measuring cell viability. Bupivacaine significantly decreased cell viability of Neuro2a cells in a time-dependent manner (*P* < 0.05 at 1 h, *P* < 0.01 at 3 h, and *P* < 0.001 at 6 h and later; Figure 1A). Treatment with 2 mM bupivacaine significantly activated caspase-3 from at time points from 1 to 9 h (*P* < 0.05 at 1 h and *P* < 0.001 thereafter; Figure 1B) and induced DNA fragmentation in Neuro2a cells (Figure 1C). 

As shown in Figure 1D, after treatment with 2 mM bupivacaine, intracellular ROS levels in Neuro2a increased significantly in a time-dependent manner at time points from 0.5 to 9 h (*P* < 0.01 at 0.5 h and *P* < 0.001 thereafter). To determine whether p38 MAPK is activated in bupivacaine-treated Neuro2a cells, protein levels of p38 and phospho-p38 were determined by Western blotting and expressed as the phospho-p38/p38 ratio. Bupivacaine-induced phospho-p38 protein expression significantly at time points from 0.5 to 3 h (*P* < 0.05); the increase in phospho-p38 levels was not statistically significant at later time points (Figure 1E). In addition, phospho-p38 protein expression was also significantly increased at time points from 0.5 to 3 h when ROS levels were induced in Neuro2a cells by cellular exposure to H_2_O_2_ (*P* < 0.05, Figure 1E).

### Bupivacaine increases WDR35 expression in Neuro2a cells

**Figure 2:**
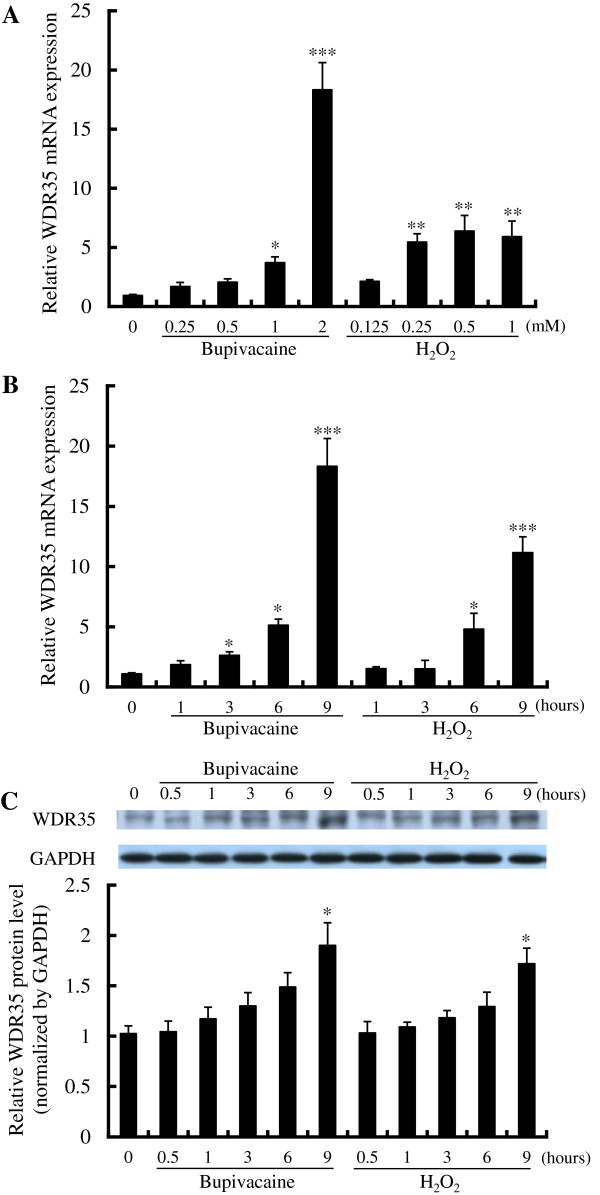
**Effect of bupivacaine or H_**2**_O_**2 **_on WDR35 expression.** (**A**) Neuro2a cells were treated with various concentrations of bupivacaine or H_2_O_2_ for 9 h. WDR35 mRNA expression was analyzed by qPCR and expressed relative to the expression of GAPDH mRNA. (**B**) WDR35 mRNA expression and (**C**) WDR35 protein expression were measured after treatment of Neuro2a cells with 2 mM bupivacaine or 0.5 mM H_2_O_2_ for the time indicated. *P < 0.05, **P < 0.01 and ***P < 0.001 vs. control (not treated with bupivacaine or H_2_O_2_, n = 6).

To determine whether WDR35 is upregulated in bupivacaine-treated Neuro2a cells, the expression of WDR35 mRNA and protein was analyzed by qPCR and Western blotting, respectively. As shown in Figure 2A, bupivacaine significantly increased WDR35 mRNA expression in a dose-dependent manner, with 2 mM bupivacaine producing an 18.1-fold increase in WDR35 mRNA over the control level (*P* < 0.001). Treatment of Neuro2a cells with 2 mM bupivacaine for 3 to 9 h significantly increased WDR35 mRNA expression (*P* < 0.05 at 3 and 6 h and *P* < 0.001 at 9 h; Figure 2B). The maximal effect was reached at 9 h, when there was an 18.3-fold increase in WDR35 mRNA over the control level. WDR35 protein expression was significantly increased in cells treated with bupivacaine for 9 h (*P* < 0.05, Figure 2C). In control experiments, treatment of Neuro2a cells with the sodium channel blocker tetrodotoxin did not alter WDR35 mRNA expression (data not shown), suggesting that the upregulation of WDR35 expression in bupivacaine-treated cells was not mediated by sodium channel blockade. 

In addition, H_2_O_2_ significantly increased WDR35 mRNA expression in a dose-dependent manner (*P* < 0.01, Figure 2A). Treatment of cells with 0.5 mM H_2_O_2_ significantly increased WDR35 mRNA expression in a time-dependent manner (*P* < 0.05 at 6 h and *P* < 0.001 at 9 h; Figure 2B). Expression of WDR35 protein was significantly increased at 9 h (*P* < 0.05, Figure 2C).

### Antioxidant EUK-8 and p38 MAPK inhibitor attenuate bupivacaine-induced cell death

**Figure 3:**
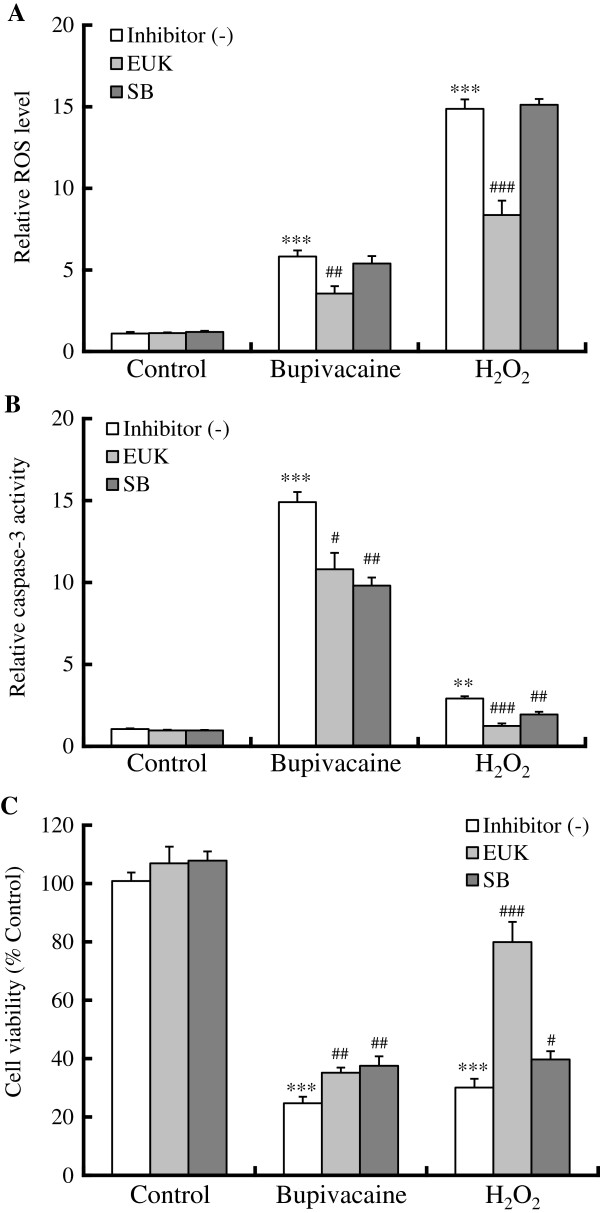
**Effect of antioxidant and p38 MAPK inhibitor on bupivacaine- or H_**2**_**O**_**2**_-induced ROS level, caspase-3 activity and cell viability.** Neuro2a cells were treated with EUK-8 (EUK, 100 μM) or SB202190 (SB, 10 μM) for 1 h, followed by bupivacaine (2 mM) or H_2_O_2_ (0.5 mM) for 9 h. (**A**) Intracellular levels of ROS, (**B**) caspase-3 activity, and (**C**) cell viability were measured (n = 6). **P < 0.01 and ***P < 0.001 vs. control (not treated with bupivacaine or H_2_O_2_), ^#^*P* < 0.05, ^##^*P* < 0.01 and ^###^*P* < 0.001 vs. absence of EUK-8 or SB202190.

EUK-8, a synthetic catalytic free radical scavenger, has protective effects in numerous models of disease processes associated with oxidative stress, including inflammation, cardiovascular diseases and neurological disorders [17-19]. To determine whether ROS generation and p38 MAPK activation are involved in bupivacaine-induced apoptosis, the effects of EUK-8 and SB202190 on ROS levels, caspase-3 activity and cell viability were measured. Neuro2a cells were treated with EUK-8 (100 μM; Calbiochem, La Jolla, CA, USA) and/or SB202190 (10 μM; Calbiochem) for 1 h, followed by bupivacaine (2 mM) for 9 h. EUK-8 treatment significantly attenuated the bupivacaine-induced increase in intracellular ROS levels (*P* < 0.01), whereas SB202190 treatment did not (Figure 3A). As shown in Figure 3B and C, both EUK-8 treatment and SB202190 treatment significantly attenuated the bupivacaine-induced increase in caspase-3 activity (*P* < 0.05 and *P* < 0.01) and cell death (*P* < 0.01). 

Similarly, treatment with EUK-8 significantly attenuated the H_2_O_2_-induced increase in intracellular ROS levels (*P* < 0.001; Figure 3A), and both EUK-8 treatment and SB202190 treatment significantly attenuated the H_2_O_2_-induced increase in caspase-3 activity (*P* < 0.01 and *P* < 0.001; Figure 3B) and cell death (*P* < 0.05 and *P* < 0.001; Figure 3C). The combination of EUK-8 and SB202190 showed no apparent additive effects or synergism (data not shown).

### Antioxidant EUK-8 and p38 MAPK inhibitor attenuate bupivacaine-induced increase in WDR35 expression

**Figure 4:**
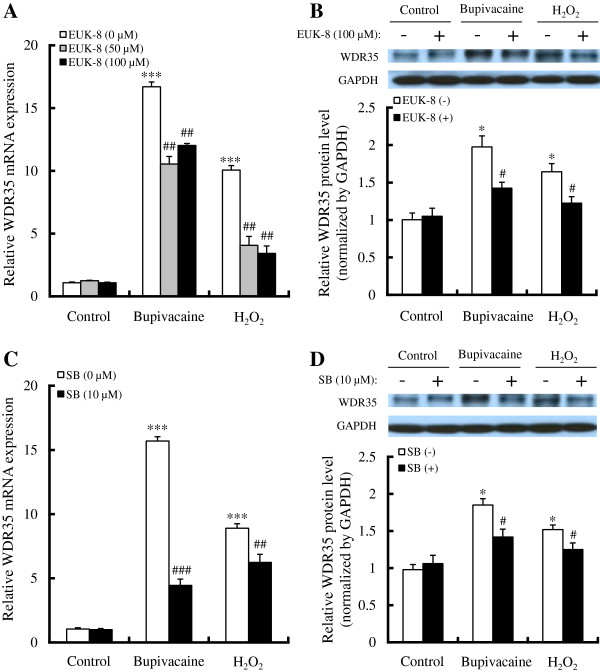
**Effect of antioxidant and p38 MAPK inhibitor on bupivacaine- or H_**2**_**O**_**2**_-induced WDR35 expression.** (**A**) Neuro2a cells were treated with various concentrations of EUK-8 for 1 h, followed by bupivacaine (2 mM) or H_2_O_2_ (0.5 mM) for 9 h (n = 6). (**B**) WDR35 protein expression with or without 100 μM EUK-8 was analyzed by Western blotting (n = 4). *P < 0.05 and ***P < 0.001 vs. control (not treated with bupivacaine or H_2_O_2_), ^#^*P* < 0.05 and ^##^*P* < 0.01 vs. absence of EUK-8. (**C**) Cells were treated with 10 μM SB202190 (SB) for 1 h, followed by bupivacaine (2 mM) or H_2_O_2_ (0.5 mM) for 9 h (n = 6). (**D**) WDR35 protein expression with or without SB202190 was analyzed by Western blotting (n = 4). *P < 0.05 and ***P < 0.001 vs. control (not treated with bupivacaine or H_2_O_2_), ^#^*P* < 0.05, ^##^*P* < 0.01 and ^###^*P* < 0.001 vs. absence of SB202190.

Neuro2a cells were treated with EUK-8 for 1 h, followed by bupivacaine (2 mM) for 9 h. As shown in Figure 4A and B, EUK-8 significantly attenuated the bupivacaine-induced increase in expression of WDR35 mRNA (*P* < 0.01) and protein (*P* < 0.05). 

In order to explore the relationship between WDR35 expression and p38 MAPK activation, Neuro2a cells were treated for 1 h with the p38 MAPK inhibitor SB202190 (10 μM), followed by bupivacaine (2 mM) for 9 h. As shown in Figure 4C and D, SB202190 significantly attenuated the bupivacaine-induced increase in expression of WDR35 mRNA (*P* < 0.001) and protein (*P* < 0.05).

In addition, treatment with EUK-8 and SB202190 significantly attenuated the H_2_O_2_-induced increase in expression of WDR35 mRNA (*P* < 0.01; Figure 4A and C) and protein (*P* < 0.05; Figure 4B and D). The combination of EUK-8 and SB202190 showed no apparent additive effects or synergism (data not shown).

### WDR35 siRNA does not inhibit the increase in caspase-3 activity and cell death in Neuro2a cells

**Figure 5:**
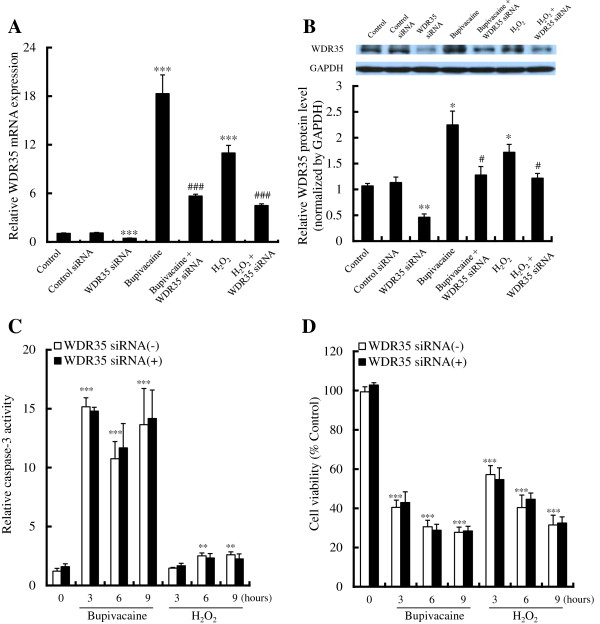
**Effect of WDR35 siRNA on bupivacaine- or H_**2**_**O**_**2**_-induced WDR35 expression, caspase-3 activity, and cell viability.** Neuro2a cells were transfected with WDR35 siRNA (5 nM) for 24 h. Bupivacaine (2 mM) or H_2_O_2_ (0.5 mM) was then added. (**A**) WDR35 mRNA expression and (**B**) WDR35 protein expression were measured after treatment of Neuro2a cells with bupivacaine or H_2_O_2_ for 9h. (**C**) Caspase-3 activity and (**D**) cell viability were measured after treatment of Neuro2a cells with bupivacaine or H_2_O_2_ for the time indicated. *P < 0.05, **P < 0.01 and ***P < 0.001 vs. control (not treated with siRNA, bupivacaine, or H_2_O_2_), ^#^*P* < 0.05 and ^###^*P* < 0.001 vs. treated with bupivacaine or H_2_O_2_ alone (n = 4-6).

Finally, in order to examine the effects of WDR35 siRNA in bupivacaine-treated Neuro2a cells, cells were transfected with WDR35 siRNA (5 nM) for 24 h, then bupivacaine (2 mM) or H_2_O_2_ (0.5 mM) was added and the cells were incubated for another 9 h. Although transfection of Neuro2a cells with WDR35 siRNA attenuated the bupivacaine- and H_2_O_2_-induced increase in expression of WDR35 mRNA (*P* < 0.001; Figure 5A) and protein (*P* < 0.05; Figure 5B), it did not inhibit the increase in caspase-3 activity (Figure 5C) or the increase in cell death (Figure 5D). Control-siRNA had no effect on the expression of WDR35 mRNA and protein and on caspase-3 activation in bupivacaine- or H_2_O_2_-treated Neuro2a cells. 

## Discussion

In this study we showed that bupivacaine induced caspase-3 activation and DNA fragmentation, indicating the occurrence of apoptosis, in Neuro2a cells. The p38 MAPK signaling pathway has been implicated in apoptosis occurring in response to distinct stimuli, such as ROS [20]. Excessive production of ROS can activate p38 MAPK, eventually causing apoptosis [6]. Bupivacaine has previously been shown to increase intracellular ROS levels in Schwann cells [7] and SH-SY5Y cells [9]. In the present study, we examined whether bupivacaine induces ROS in Neuro2a cells. We found that bupivacaine significantly and time-dependently elevated intracellular ROS levels in Neuro2a cells. H_2_O_2_ also elevated ROS levels in Neuro2a cells. We then confirmed the activation of p38 MAPK in cells treated with bupivacaine or H_2_O_2_. Our results are in agreement with recent reports that bupivacaine elicits ROS production and induces apoptosis accompanied by activation of p38 MAPK in other cell types [8,9]. Further, EUK-8 significantly attenuated the bupivacaine-induced increase in intracellular ROS levels, caspase-3 activity and cell death. In contrast, SB202190 attenuated the bupivacaine-induced increase in caspase-3 activity and cell death, but had no effect on ROS levels. Effects on H_2_O_2_-induced increases in intracellular ROS levels, caspase-3 activity and cell death were similar. These results indicate that bupivacaine-induced ROS generation may be upstream of p38 MAPK activation, leading to apoptosis in Neuro2a cells.

The family of WD40-repeat proteins comprises a large number of proteins and is involved in a wide variety of cellular processes such as signal transduction, cell growth, proliferation, and apoptosis [10,11]. WDR35 encodes a novel member of this protein family. In a mouse mutation screen for developmental phenotypes, Mill et al. [12] identified a mutation in the WDR35 gene as a cause of midgestation lethality associated with abnormalities characteristic of defects in the Hedgehog signaling pathway. More recently, clinical studies have identified relationships between the WDR35 gene and coronary artery disease [21] and Sensenbrenner syndrome [22]. On the cellular level, we reported that WDR35 siRNA inhibited both TNF-α − induced caspase-3 activation and apoptosis in HEK293 cells [13]. More recently, we reported that WDR35 elicits an inhibitory effect on the anti-apoptotic proteins Bcl-2 and Bcl-xL in hepatocytes treated with LPS, causing release of cytochrome *c* from mitochondria and activation of caspase-3, resulting in LPS-induced hepatocyte apoptosis [14]. These results indicate that WDR35 is involved in apoptosis mediated by caspase-3 activation via both the death receptor and mitochondrial signaling pathways.

In order to determine whether WDR35 is involved in bupivacaine-induced apoptosis, we examined the expression of WDR35 in bupivacaine-treated Neuro2a cells. In the present study, we showed that WDR35 expression significantly increased with respect to both mRNA and protein levels. These results are consistent with our previous reports of enhanced expression of WDR35 in the kidneys of streptozotocin-induced diabetic rats [23] and in the livers of LPS-treated rats [14]. Further, we examined the effects of EUK-8 and SB202190 on WDR35 expression in bupivacaine-treated Neuro2a cells. These compounds significantly attenuated the bupivacaine-induced increase in WDR35 expression. Collectively, we provided the first evidence that bupivacaine-induced WDR35 expression may be downstream of ROS generation and subsequent p38 MAPK activation.

In our previous studies, WDR35 siRNA inhibited increases in caspase-3 activity in HEK293 cells induced by TNF-α [13], in hepatocytes induced by LPS [14], and in NRK52E cells induced by high glucose [23]. Interestingly, we found that blocking upregulation of WDR35 expression with WDR35 siRNA in Neuro2a cells had no effect on the increase in caspase-3 activity or the increase in cell death induced by bupivacaine. The lack of WDR35 involvement in caspase-3 activation in bupivacaine-induced apoptosis in Neuro2a cells was unexpected. It is difficult to clearly explore the discrepancy in results between the previous studies and the present study because available information relating to physiological role of WDR35 is very limited. Nevertheless, our findings may paradoxically suggest the existence of another mechanism of bupivacaine-induced apoptosis independent from WDR35 expression in Neuro2a cells. In fact, several lines of evidence indicated that bupivacaine-induced apoptosis has multiple mechanisms with depending on the types of cell. Bupivacaine induces neurotoxicity through activation of the AMP-activated protein kinase (AMPK)-dependent pathway in Schwann cells [24] and SH-SY5Y cells [25], and the extracellular signal-regulated kinase (ERK)-dependent pathway in Neuro2a cells [26]. These findings raise an interesting possibility that some, if not all, of these pathways could modulate bupivacaine-induced neurotoxicity independently or collaboratively. We will perform further studies using caspase-3 inhibitors, other cell types and other apoptotic stimuli to investigate the sequence of events.

## Conclusions

In conclusion, our results indicate that bupivacaine induces apoptosis in Neuro2a cells. Bupivacaine induces ROS generation and p38 MAPK activation, resulting in an increase in WDR35 expression. However, the increase in WDR35 expression doesn’t seem to be involved in bupivacaine-induced apoptosis in Neuro2a cells. These results may suggest the existence of another mechanism of bupivacaine-induced apoptosis independent from WDR35 expression in Neuro2a cells.

## Methods

### Cell culture

Mouse neuroblastoma Neuro2a cells were purchased from the Health Science Research Resources Bank (Tokyo, Japan). The cells were maintained in RPMI-1640 medium (Sigma-Aldrich, St. Louis, MO, USA) containing 10% fetal bovine serum with 100 units/ml penicillin and 100 g/ml streptomycin (Gibco BRL, Grand Island, NY, USA). The cells were maintained at 37°C in a humidified atmosphere with 5% CO_2_. The culture medium was replaced every 2–3 days. To prepare cell suspensions, the cells were treated with trypsin (0.25%)-EDTA (1 mM) (Gibco BRL, Grand Island, NY, USA), transferred to a 6-cm culture plate at a density of 1 × 10^6^ cells per dish, and cultured overnight.

### Measurement of cell viability using the MTT assay

Neuro2a cells were placed at a density of 1 × 10^4^ cells per well in a 96-well cell culture plate, and cell viability was determined with a cell proliferation kit (Roche Applied Sciences, Mannheim, Germany) according to the manufacturer’s instructions. Briefly, after exposure of the cells to 2 mM bupivacaine for a period of 1 to 12 h, 10 μl 3-[4,5-dimethylthiazol-2-yl]-2,5-diphenyl tetrazolium bromide (MTT) was added to each well, and the cells were incubated at 37°C for 4 h. The supernatants were aspirated carefully and 100 μl of solubilization buffer (10% SDS in 0.01 M HCl) was added to each well. The absorbance at 550 nm was measured with a microplate reader (VersaMax, Molecular Devices, Sunnyvale, CA, USA).

### Assessment of caspase-3 activity

Caspase-3 activity in cultured Neuro2a cells was measured with a caspase-3/CPP32 fluorometric assay kit (Medical & Biological Laboratories, Nagoya, Japan) according to the manufacturer’s instructions. These assays are based on the detection of the cleavage products of a fluorometric caspase substrate for caspase-3; DEVD-AFC (Asp-Glu-Val-Asp-7-amino-4-trifluoromethyl coumarin). In brief, cells were homogenized in the cell lysis buffer supplied in the kit. Samples (200 μg protein) were then mixed with 2× reaction buffer containing 10 mM dithiothreitol (DTT). After incubation at 37°C for 2 h, free AFC that had been cleaved from the fluorometric substrate was quantified using a Fluoroskan Ascent FL microplate fluorometer (Labsystems, Helsinki, Finland) with excitation/emission (Ex/Em) of 400/505 nm, as reported previously [27].

### Analysis of DNA ladder formation

DNA fragmentation in Neuro2a cells exposed to bupivacaine was measured with an apoptotic DNA ladder assay kit (Roche Applied Sciences) according to the manufacturer’s instructions. Briefly, 200 μl of a cell suspension in PBS was mixed with 200 μl of binding buffer supplied in the kit. After incubation for 10 min at room temperature, 100 μl of isopropanol was added to the sample and mixed by vortexing. Total genomic DNA was then isolated by using glass fiber filters, treated with RNase A (400 μg/ml) at 37°C for 1 h, electrophoresed in a 1.5% agarose gel, and visualized with ethidium bromide staining under UV light.

### Measurement of ROS

The OxiSelect™ intracellular ROS assay kit (Cell Biolabs, Inc., San Diego, CA) was used according to the manufacturer’s instructions. This assay uses the cell-permeable fluorogenic probe 2^′^,7^′^-dichlorodihydrofluorescein diacetate (DCFH-DA). The fluorescence intensity is proportional to the concentration of ROS within the cell cytosol. Briefly, Neuro2a cells were placed in a clear 96-well cell culture plate (5 × 10^4^ cells per well) overnight in the incubator. The cells were then exposed to DCFH-DA in medium for 30 min. After being washed twice with PBS, the cells loaded with DCFH-DA were exposed to 2 mM bupivacaine for periods from 0.5 to 9 h. The cells were lysed by adding 100 μl of cell lysis buffer, mixing thoroughly, and incubating for 5 min at room temperature. The fluorescence was read with a fluorometric plate reader at 480/530 nm.

### Quantitative real-time polymerase chain reaction (qPCR) analysis

Total RNA (1 μg) was extracted from cultured Neuro2a cells with TRIzol® reagent (Invitrogen, Carlsbad, CA, USA) and reverse transcribed with the ReverTra Ace® qPCR RT kit (Toyobo, Osaka, Japan). qPCR was performed with the ABI StepOne Plus real-time PCR system and a TaqMan Gene Expression Assay (Applied Biosystems, Tokyo, Japan) according to the manufacturer’s instructions. The primers and TaqMan MGB probe for mouse WDR35 (Mm00552650_m1) were purchased from Applied Biosystems. The amount of WDR35 PCR product was calculated relative to the internal control glyceraldehyde-3-phosphate dehydrogenase (GAPDH, Mm99999915_g1; Applied Biosystems) and was compared between experimental and control groups by the ΔΔC_T_ method, as reported previously [13].

### Western blot analysis

Protein samples from cultured Neuro2a cells were homogenized in sample buffer [50 mM Tris–HCl, pH 6.8, 0.2 M DTT, 2% sodium dodecyl sulfate (SDS), 10% glycerol, 0.1% bromophenol blue (BPB)] containing a mixture of protease inhibitors (Complete Protease Inhibitor Cocktail, Roche Applied Sciences) and heated in boiling water for 5 min. Proteins were separated by SDS-PAGE and transferred to PVDF membranes (Immobilon-P, Millipore, Bedford, MA, USA). These membranes were probed with anti-WDR35 peptide antibody (amino acids 459–473, 1:500), which was designed, produced, and purified by Medical & Biological Laboratories (Nagoya, Japan), or with antibodies against p38 MAPK, phospho-p38 (P-p38) MAPK, or GAPDH (Cell Signaling Technology, Danvers, MA, USA; 1:1000). Detection was performed with the Western blotting reagent ECL Prime (GE Healthcare, Buckinghamshire, UK). Protein levels were quantified by densitometric scanning with the Gel-Pro Analyzer (Media Cybernetics, Inc., USA) and expressed as the ratio to GAPDH levels as described previously [23].

### Small interfering RNA (siRNA) transfection

VNeuro2a cells were transfected with 5 nM WDR35-specific siRNA (siRNA ID: s93029; Ambion, Austin, TX, USA) or 10 nM control siRNA (negative control #1 siRNA, catalog no. 4390843; Ambion) using Lipofectamine RNAiMAX (Invitrogen) according to the manufacturer’s instructions. Effects of WDR35 siRNA on the expression of WDR35 mRNA were tested with qPCR after 24 h of transfection. In order to examine the effect of WDR35 siRNA on bupivacaine- or H_2_O_2_-induced apoptosis, after 24 h of transfection with 5 nM WDR35 siRNA, cells were further incubated with bupivacaine (2 mM) or H_2_O_2_ (0.5 mM) for 3, 6, or 9 h, and caspase-3 activation was then examined in these cells.

### Statistical analysis

All results were expressed as the mean ± standard error of the mean (SEM). Data were analyzed with one-way analysis of variance (ANOVA), and significant differences between treatments were assessed by use of Tukey’s test. Differences were considered significant at *P* < 0.05.

## Competing interests

The authors declare that they have no competing interests.

## Authors’ contributions

MH, LH, FK and SO conceptualized the study design, were involved in data analysis, coordinated all aspects of the study, wrote the manuscript and critically appraised the manuscript. KT, GGF and JHF performed the cell experiments and contributed to data acquisition and analysis. NI and YF were involved in the conceptualization of the study, data analysis and acquisition, in addition to contributing to critical appraisal and writing of the manuscript. All authors read and approved the final manuscript.

## References

[B1] Perez-Castro R Patel S Garavito-Aguilar ZV Rosenberg A Recio-Pinto E Zhang J Blanck TJ Xu F Cytotoxicity of local anesthetics in human neuronal cells Anesth Analg 2009 108 997 1007 10.1213/ane.0b013e31819385e1 19224816

[B2] Auroy Y Narchi P Messiah A Litt L Rouvier B Samii K Serious complications related to regional anesthesia: results of a prospective survey in France Anesthesiology 1997 87 479 486 10.1097/00000542-199709000-00005 9316950

[B3] Radwan IA Saito S Goto F The neurotoxicity of local anesthetics on growing neurons: a comparative study of lidocaine, bupivacaine, mepivacaine, and ropivacaine Anesth Analg 2002 94 319 324 11812691 10.1097/00000539-200202000-00016

[B4] Werdehausen R Fazeli S Braun S Hermanns H Essmann F Hollmann MW Bauer I Stevens MF Apoptosis induction by different local anaesthetics in a neuroblastoma cell line Br J Anaesth 2009 103 711 718 10.1093/bja/aep236 19700777

[B5] Cuadrado A Nebreda AR Mechanisms and functions of p38 MAPK signalling Biochem J 2010 429 403 417 10.1042/BJ20100323 20626350

[B6] Harper SJ LoGrasso P Signalling for survival and death in neurones: the role of stress-activated kinases, JNK and p38 Cell Signal 2001 13 299 310 10.1016/S0898-6568(01)00148-6 11369511

[B7] Park CJ Park SA Yoon TG Lee SJ Yum KW Kim HJ Bupivacaine induces apoptosis via ROS in the Schwann cell line J Dent Res 2005 84 852 857 10.1177/154405910508400914 16109997

[B8] Lirk P Haller I Colvin HP Lang L Tomaselli B Klimaschewski L Gerner P In vitro, inhibition of mitogen-activated protein kinase pathways protects against bupivacaine- and ropivacaine-induced neurotoxicity Anesth Analg 2008 106 1456 1464 10.1213/ane.0b013e318168514b 18420860

[B9] Lu J Xu SY Zhang QG Xu R Lei HY Bupivacaine induces apoptosis via mitochondria and p38 MAPK dependent pathways Eur J Pharmacol 2011 657 51 58 10.1016/j.ejphar.2011.01.055 21315711

[B10] Neer EJ Schmidt CJ Nambudripad R Smith TF The ancient regulatory-protein family of WD-repeat proteins Nature 1994 371 297 300 10.1038/371297a0 8090199

[B11] Smith TF Gaitatzes C Saxena K Neer EJ The WD repeat: a common architecture for diverse functions Trends Biochem Sci 1999 24 181 185 10.1016/S0968-0004(99)01384-5 10322433

[B12] Mill P Lockhart PJ Fitzpatrick E Mountford HS Hall EA Reijns MA Keighren M Bahlo M Bromhead CJ Budd P Aftimos S Delatycki MB Savarirayan R Jackson IJ Amor DJ Human and mouse mutations in WDR35 cause short-rib polydactyly syndromes due to abnormal ciliogenesis Am J Hum Genet 2011 88 508 515 10.1016/j.ajhg.2011.03.015 21473986 PMC3071922

[B13] Feng GG Li C Huang L Tsunekawa K Sato Y Fujiwara Y Komatsu T Honda T Fan JH Goto H Koide T Hasegawa T Ishikawa N Naofen, a novel WD40-repeat protein, mediates spontaneous and tumor necrosis factor-induced apoptosis Biochem Biophys Res Commun 2010 394 153 157 10.1016/j.bbrc.2010.02.133 20193664

[B14] Fan JH Feng GG Huang L Tsunekawa K Honda T Katano Y Hirooka Y Goto H Kandatsu N Ando K Fujiwara Y Koide T Okada S Ishikawa N Role of naofen in apoptosis of hepatocytes induced by lipopolysaccharide through mitochondrial signaling in rats Hepatol Res 2012 42 696 705 10.1111/j.1872-034X.2012.00972.x 22409254

[B15] Unami A Shinohara Y Ichikawa T Baba Y Biochemical and microarray analyses of bupivacaine-induced apoptosis J Toxicol Sci 2003 28 77 94 10.2131/jts.28.77 12820540

[B16] Werdehausen R Braun S Essmann F Schulze-Osthoff K Walczak H Lipfert P Stevens MF Lidocaine induces apoptosis via the mitochondrial pathway independently of death receptor signaling Anesthesiology 2007 107 136 143 10.1097/01.anes.0000268389.39436.66 17585225

[B17] Gonzalez PK Zhuang J Doctrow SR Malfroy B Benson PF Menconi MJ Fink MP EUK-8, a synthetic superoxide dismutase and catalase mimetic, ameliorates acute lung injury in endotoxemic swine J Pharmacol Exp Ther 1995 275 798 806 7473169

[B18] Bruce AJ Malfroy B Baudry M Beta-Amyloid toxicity in organotypic hippocampal cultures: protection by EUK-8, a synthetic catalytic free radical scavenger Proc Natl Acad Sci USA 1996 93 2312 2316 10.1073/pnas.93.6.2312 8637869 PMC39792

[B19] Kawakami S Matsuda A Sunagawa T Noda Y Kaneko T Tahara S Hiraumi Y Adachi S Matsui H Ando K Fujita T Maruyama N Shirasawa T Shimizu T Antioxidant, EUK-8, prevents murine dilated cardiomyopathy Circ J 2009 73 2125 2134 10.1253/circj.CJ-09-0204 19749480

[B20] Kennedy KA Sandiford SD Skerjanc IS Li SS Reactive oxygen species and the neuronal fate Cell Mol Life Sci 2012 69 215 221 10.1007/s00018-011-0807-2 21947442 PMC11114775

[B21] Lu X Wang L Chen S He L Yang X Shi Y Cheng J Zhang L Gu CC Huang J Wu T Ma Y Li J Cao J Chen J Ge D Fan Z Li Y Zhao L Li H Zhou X Chen L Liu D Chen J Duan X Hao Y Wang L Lu F Liu Z Yao C Genome-wide association study in Han Chinese identifies four new susceptibility loci for coronary artery disease Nat Genet 2012 44 890 894 10.1038/ng.2337 22751097 PMC3927410

[B22] Bacino CA Dhar SU Brunetti-Pierri N Lee B Bonnen PE WDR35 mutation in siblings with Sensenbrenner syndrome: A ciliopathy with variable phenotype Am J Med Genet A 2012 158 2917 2924 22987818 10.1002/ajmg.a.35608 PMC4000731

[B23] Sato Y Feng GG Huang L Fan JH Li C An J Tsunekawa K Kurokawa S Fujiwara Y Komatsu T Kondo F Ishikawa N Enhanced expression of naofen in kidney of streptozotocin-induced diabetic rats: possible correlation to apoptosis of tubular epithelial cells Clin Exp Nephrol 2010 14 205 212 10.1007/s10157-010-0276-1 20224876

[B24] Lee SJ Shin TJ Kang IS Ha JH Lee SC Kim HJ AMPK attenuates bupivacaine-induced neurotoxicity J Dent Res 2010 89 797 801 10.1177/0022034510366823 20448244

[B25] Lu J Xu SY Zhang QG Lei HY Bupivacaine induces reactive oxygen species production via activation of the AMP-activated protein kinase-dependent pathway Pharmacology 2011 87 121 129 10.1159/000323402 21304223

[B26] Wang Z Shen J Wang J Lu T Li C Zhang X Liu L Ding Z Lithium attenuates bupivacaine-induced neurotoxicity in vitro through phosphatidylinositol-3-kinase/threonine-serine protein kinase B- and extracellular signal-regulated kinase-dependent mechanisms Neuroscience 2012 206 190 200 22233781 10.1016/j.neuroscience.2011.12.043

[B27] Huang L Hotta Y Miyazeki K Ishikawa N Miki Y Sugimoto Y Yamada J Nakano A Hishiwaki K Shimada Y Different effects of optical isomers of the 5-HT_1A_ receptor antagonist pyrapyridolol against postischemic guinea-pig myocardial dysfunction and apoptosis through the mitochondrial permeability transition pore Eur J Pharmacol 2006 534 165 177 10.1016/j.ejphar.2006.01.040 16612842

